# Hazardous Effects of SiO_2_ Nanoparticles on Liver and Kidney Functions, Histopathology Characteristics, and Transcriptomic Responses in Nile Tilapia (*Oreochromis niloticus*) Juveniles

**DOI:** 10.3390/biology10030183

**Published:** 2021-03-02

**Authors:** Hany M.R. Abdel-Latif, Mustafa Shukry, Omnia I. El Euony, Mohamed Mohamed Soliman, Ahmed E. Noreldin, Hanan A. Ghetas, Mahmoud A.O. Dawood, Mohamed A. Khallaf

**Affiliations:** 1 Department of Poultry and Fish Diseases, Faculty of Veterinary Medicine, Alexandria University, Alexandria 22758, Egypt; 2 Department of Physiology, Faculty of Veterinary Medicine, Kafrelsheikh University, Kafrelsheikh 33516, Egypt; mostafa.ataa@vet.kfs.edu.eg; 3 Department of Forensic Medicine and Toxicology, Faculty of Veterinary Medicine, Alexandria University, Alexandria 22758, Egypt; omnia.ismail@alexu.edu.eg; 4 Clinical Laboratory Sciences Department, Turabah University College, Taif University, P.O. Box 11099, Taif 21944, Saudi Arabia; mmsoliman@tu.edu.sa; 5 Histology and Cytology Department, Faculty of Veterinary Medicine, Damanhour University, Damanhour 22511, Egypt; ahmed.elsayed@damanhour.edu.eg; 6 Department of Aquatic Animal Medicine and Management, Faculty of Veterinary Medicine, University of Sadat City, Sadat City 32897, Egypt; hanan.ghetas@vet.usc.edu.eg (H.A.G.); mohamed.khallaf@vet.usc.edu.eg (M.A.K.); 7 Department of Animal Production, Faculty of Agriculture, Kafrelsheikh University, Kafrelsheikh 33516, Egypt; Mahmoud.dawood@agr.kfs.edu.eg

**Keywords:** Nile tilapia, SiO_2_NPs, hepato-renal functions, histopathology, transcriptomic profile

## Abstract

**Simple Summary:**

Waterborne exposure of Nile tilapia (*Oreochromis niloticus*) juveniles to sub-lethal concentrations of silicon dioxide nanoparticles (SiO_2_NPs) induced hepato-renal damage through elevation of aspartate transaminase (AST), alanine transaminase (ALT), and alkaline phosphatase (ALP) activities as well as creatinine and blood urea levels. SiO_2_NPs induced irreversible dose-dependent histopathological changes in the hepatopancreas, gills, and posterior kidneys, alongside modulation of the pro-inflammatory cytokines, apoptosis-related genes, and oxidative stress genes in gills and liver of exposed fish.

**Abstract:**

The current investigation assessed the impacts of sub-lethal concentrations of silicon dioxide nanoparticles (SiO_2_NPs) on hepato-renal functions, histopathological characteristics, and gene transcription in gills and liver of Nile tilapia juveniles. Fish were exposed to 20, 40, and 100 mg/L of SiO_2_NPs for 3 weeks. Pairwise comparisons with the control group showed a significant dose-dependent elevation in serum ALP, ALT, and AST enzyme activities as well as blood urea and creatinine levels in SiO_2_NP-intoxicated groups. Exposure to 100 mg/L SiO_2_NPs significantly upregulated expression of *HSP70*, *TNF-α*, *IL-1β*, and *IL-8* genes in the gills as compared to the control group. Moreover, exposure to 100 mg/L SiO_2_NPs significantly upregulated the expression *SOD*, *HSP70*, *IL-1β*, *IL-8*, and *TNF-α* genes in the hepatic tissues as compared to the control group. Exposure of fish to 20 mg SiO_2_NPs/L significantly increased the mRNA expression levels of *IL-12* in both the gills and liver tissues. Notably, all tested SiO_2_NP concentrations significantly upregulated the transcription of *CASP3* gene in gills and liver of Nile tilapia as compared to the control group. Interestingly, varying histopathological alterations in renal, hepatopancreatic, and branchial tissues were observed to be correlated to the tested SiO_2_NP concentrations. In conclusion, our results provide additional information on the toxic impacts of SiO_2_NPs in Nile tilapia at the hematological, tissue, and molecular levels.

## 1. Introduction

Globally, engineered nanomaterials (ENMs) are increasingly being manufactured due to their wide range of applications in various industrial products [1]. However, the misuse and unhygienic disposal of ENMs may lead to their release into aquatic ecosystems, which can negatively pose serious risk problems to human beings [2,3] and living aquatic biota including fish and bivalve mollusks [4,5,6].

Among ENMs, silica nanostructure or silicon dioxide nanoparticles (SiO_2_NPs) have gained great importance due to their unique beneficial uses for various biomedical purposes such as ultrasound imaging, cell labeling, and drug delivery [7,8]. Moreover, SiO_2_NPs are widely used for various agricultural purposes [9] and in industrial processes such as coatings and paintings [10], printing toners and polishing materials [11], and in medicinal and analytical applications [12]. Despite their aforementioned beneficial uses, the emergence of SiO_2_NPs in the aquatic environment can induce serious toxicological consequences. In this regard, investigations showed the toxic impacts of SiO_2_NPs on various fish species. For instance, exposure of Mozambique tilapia (*Oreochromis mossambicus*) to sub-lethal doses of SiO_2_NPs provoked the occurrence of oxidative stress and irreversible alterations in branchial and hepatic tissues [13,14], changes in hepatic transaminases and histopathological characteristics [15,16], and damage to the renal histological structures [17]. SiO_2_NPs negatively altered hematological indices, plasma ion regulation, and Na^+^/K^+^ ATPase activities in the gills of rohu (*Labeo rohita*) [18]. Moreover, reports in zebrafish (*Danio rerio*) showed that SiO_2_NPs caused DNA fragmentation and alterations to antioxidant enzymatic mechanisms [19], had toxic effects on embryonic development [20,21], and led to oxidative stress [22,23] and behavioral neurotoxicity [24].

To date, insufficient information has been available with regard to the toxic impacts of sub-lethal concentrations of SiO_2_NPs in Nile tilapia. Thus, this study sheds light on the toxicological impacts of SiO_2_NPs in Nile tilapia at the hematological, histopathological, and molecular levels. Herein, we have evaluated the effects of sub-chronic toxicity with different doses of SiO_2_NPs with regard to hepato-renal functions, histopathological alterations (gills, liver, and posterior kidney), and tissue transcriptomic analytics.

## 2. Materials and Methods

### 2.1. Silicon Dioxide Nanoparticles (SiO_2_NPs)

#### 2.1.1. Preparation and Characterization Methods

SiO_2_NPs were purchased from Naqaa Nanotechnology Co., Cairo, Egypt, and were synthesized and prepared following Stöber’s method [25]. Appropriate quantities of ethanol, tetraethoxyorthosilicate, and distilled water were continuously stirred for 30 min. To this solution the required amount of ammonia was added to adjust the pH to 9. Then, the reaction mixture was continuously stirred for 48 h at room temperature. A white-colored precipitate solution was obtained. Then, this solution was ultra-centrifuged at 8000 rpm for 15 min. After centrifugation, drying was carried out, resulting in pure white SiO_2_NPs [18].

The morphology and particle size of SiO_2_NPs was determined at 120 KV by transmission electron microscopy (TEM) (JEM-1400, TEM, JEOL Ltd., Tokyo, Japan). Surface characterization of the synthesized SiO_2_NPs was accomplished using scanning electron microscopy (SEM) and energy-dispersive X-ray spectroscopy (EDX) (JSM-5300, JEOL Ltd., Tokyo, Japan). TEM, SEM, and EDX procedures were performed at the Electronic Microscope Unit (Faculty of Science, Alexandria University, Egypt). The zeta potentials of SiO_2_NPs in solution were evaluated using a Zetasizer Nano Series apparatus (Model 1801102S, Malvern Instruments, UK) at the Central Laboratory, Faculty of Pharmacy, Alexandria University, Alexandria, Egypt.

#### 2.1.2. SiO_2_NP Stock Solution

SiO_2_NP stock solution was formulated through the dispersion of the NPs into ultra-pure water (Milli-Q type 1 Ultrapure Water Purification Systems) (Millipore Co., Billerica MA, USA); then, sonication was performed for 1 h in a bath-type sonicator (50 W/L, 40 kHz). The sonication process was performed 30 min before daily dosing in the experiments. During the experimental periods, the test solution in each experimental group was replenished daily to maintain the relative constant concentrations and dispersity of SiO_2_NPs.

### 2.2. Animal Ethics

The experimental procedures in the current paper were demonstrated in agreement with the guidelines issued by the Local Experimental Animal Care Committee and permitted by the Institutional Ethics Committee of Faculty of Veterinary Medicine, Alexandria University, Alexandria, Egypt (Approval No. Alex-202345).

### 2.3. Fish Procurement, Acclimation, and Rearing

Nile tilapia (*Oreochromis niloticus*) juveniles (average length 6–7 cm, and initial body weight 15.50 ± 0.5 g) were procured from Saft Khalid fish hatchery (Behera Province, Egypt) and were then conveyed to the wet laboratory. Fish were kept for two weeks in 500-L tanks in order to become acclimatized to the laboratory environment. During acclimation and throughout the whole experimental period, fish were fed ad libitum with a well-balanced commercially purchased diet (30% crude protein) (Aller Aqua Co., October City, Egypt) and met all the requirements sufficient for optimum growth of Nile tilapia [26]. During the experiments, fish were reared in glass aquaria (70 × 40 × 35 cm) supported with fresh dechlorinated tap water supplied with compressed air via air stones using air pumps. The light was adjusted with a 12 h:12 h light and dark cycle by fluorescent light tubes. To ensure a safe and healthy environment, daily siphoning of one third (1/3) of the water per aquarium was done to reduce the contamination from the uneaten food and metabolic waste. This was exchanged with new water obtained from the storage tank.

### 2.4. Water Quality Parameters

Water quality parameters were assessed daily prior to daily SiO_2_NP dosing. The physico-chemical properties of the water during the acclimation period and during the experiments conducted in the present study were maintained for dissolved oxygen (7.50 ± 0.46 mg/L), pH value (7.80 ± 0.5), temperature (28.5 ± 0.5 °C), nitrite (0.007 mg/L), total alkalinity (17.83 mg/L), total hardness (15.3 mg/L), and un-ionized ammonia (0.014 ± 0.04 mg/L) [27].

### 2.5. Fish Exposure to SiO_2_NPs and Experimental Setup

The acute toxicity (96-h exposure) test was done to estimate the median lethal dose concentrations (LC50) of SiO_2_NPs in accordance with Finney’s probit analysis [28]. According to the LC50 findings, sub-lethal levels (1/2, 1/5, and 1/10 of the 96 h LC50 value) corresponding to 100, 40, and 20 mg/L, respectively, were used for the sub-lethal toxicological study (3 weeks). One-hundred-and-twenty Nile tilapia juveniles were allocated into 4 experimental groups. Fish were randomly distributed in triplicates (*n* = 3) (each replicate contained 10 fish) to ensure the possible reproducibility of the results. Groups I, II, and III were exposed to 20, 40, and 100 mg/L of SiO_2_NP solution, respectively. Group IV was sustained in dechlorinated tape water and served as the control. The experiment was continued for 3 weeks.

### 2.6. Sampling

#### 2.6.1. Serum Samples

Before blood sampling, fish were starved for 24 h, and 3 fish from each replicate (9 fish per group) were tested. After that, fish were anesthetized by buffered tricaine methanesulphonate (MS-222) (Finquel, Argent Chemical Laboratories, Redmond, Washington, USA) (100 µg/mL); blood was sampled from the caudal veins using a 1-mL syringe. Blood samples were left to clot at room temperature for collection of sera. Sera samples were separated by centrifugation at 3000× *g* for 15 min into a centrifuge tube and then stored at −20 °C until use in serum measurements.

#### 2.6.2. Tissue Samples

After the end of the experimental study, fish were euthanized using an overdose of MS-222. Tissue specimens (9 samples/group) were collected from the liver, gills, and kidneys of the control and experimentally intoxicated fish for histopathological studies. Other liver and gill specimens (9 samples/group) were assembled, instantly frozen in liquid nitrogen, and then stored at –80 °C until use for further molecular assays.

### 2.7. Serum Biochemical Measurements

Blood urea nitrogen and serum creatinine levels were estimated using fish-specific kits (Bio diagnostic Co., Cairo, Egypt) in accordance with the methods described by Coulombe and Favreau [29] and Larsen [30], respectively. The enzyme activity of serum transaminases such as alanine transaminase (ALT) and aspartate transaminase (AST) [31], and alkaline phosphatase (ALP) [32] was determined calorimetrically using specific kits (Bio diagnostic Co., Giza, Egypt) in accordance with the manufacturer’s guidelines

### 2.8. Gene Transcription

#### 2.8.1. Extraction of RNA and Synthesis of cDNA

RNA was extracted from the liver and gill tissues (100 mg) and used for qRT-PCR. The total RNA was prepared using Trizol reagent (iNtRON Biotechnology, Inc., Seongnam, Gyeonggi-do, Korea), in line with the instructions obtained from the manufacturer. The quantity of the extracted RNA was confirmed by Nanodrop (Uv–Vis spectrophotometer Q5000/Quawell, San Jose, CA, USA). Next, complementary DNA (cDNA) was synthesized using a SensiFAST™ cDNA synthesis kit (Bioline/Meridian Bioscience, London, UK) in line with the manufacturer’s instructions. The cDNA samples were then stored at −20 °C until use.

#### 2.8.2. qRT-PCR

Table 1 shows the specific primer sequences and NCBI GenBank accession numbers of the target mRNA used in the present study, including stress-related genes (such as superoxide dismutase (*SOD*), glutathione peroxidase (*GPX*), catalase (*CAT*), and heat shock protein 70 (*HSP70*)), pro-inflammatory cytokines (such as tumor necrosis factor-alpha (*TNF-α*)), interleukins (including interleukin 1 beta (*IL-1β*), *IL-8*, *IL-12*, and *IL-10*), and caspase 3 (*CASP3*, an apoptosis-related gene). Moreover, beta-actin (*β-actin*) was operated as a housekeeping gene (a reference) to quantify mRNA expression folds in the tested tissues of Nile tilapia.

The SYBR green method was used to quantify the mRNA expression folds using qRT-PCR (SensiFast SYBR Lo-Rox kit, Bioline/Meridian Bioscience, London, UK). Conditions of thermocycler utilized for the PCR reactions were 10 min at 95 °C for one cycle (initial denaturation), followed by 35 cycles of 15 s at 95 °C (secondary denaturation), 30 min at 60 °C (annealing), and finally 85 °C for 10 min (extension). The runs were conducted in triplicates, and the mRNA expression folds were standardized to the β-actin mRNA transcripts according to the 2^−ΔΔCT^ method [33]. The quality control measures of the qPCR reaction were closely followed (please see Appendix A).

### 2.9. Histopathological Studies

Collected specimens were washed with sterile saline solution and then directly fixed in 10% formalin solution for 48 h. The paraffin embedding technique was used to process fixed specimens in accordance with Bancroft and Gamble [34]. Tissue specimens were sectioned into numerous sections (5–8 μm) by ultra-microtome (Leica Microsystems, Wetzlar, Germany), and then stained by the hematoxylin and eosin (H & E) stain [35]. Demonstrative photomicrographs were taken from the prepared tissue sections using a digital camera (Leica EC3, Leica, Germany) that joined to a microscope (Leica DM500, Germany) to evaluate the histopathological alterations.

### 2.10. Statistical Analytics

Data were presented as means ± the standard error of means (SEM). All data were testified for the normality and homogeneity of variances by Kolmogorov–Smirnov and Levene’s tests. One-way ANOVA was done and followed by Turkey’s multiple range test as a post hoc test. Statistics were done using the SPSS program (SPSS, version 22.0; SPSS Inc., Chicago, IL, USA) and GraphPad Prism Software (version 5) to evaluate the significant differences between the SiO_2_NP-exposed groups in comparison with the control group. *p* < 0.05 was considered to be statistically significant.

## 3. Results

### 3.1. SiO_2_NP Characterizations

**Figure 1:**
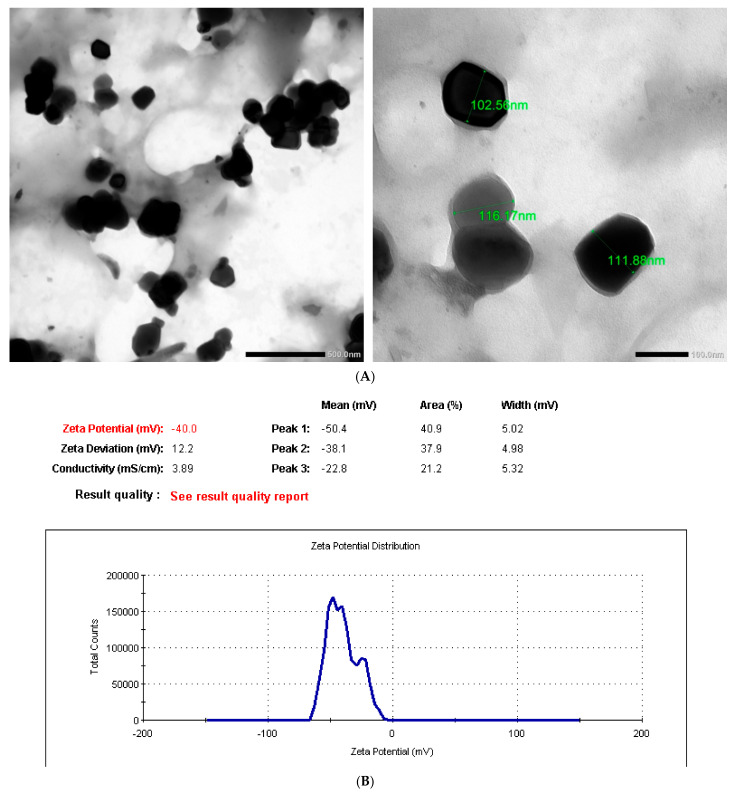

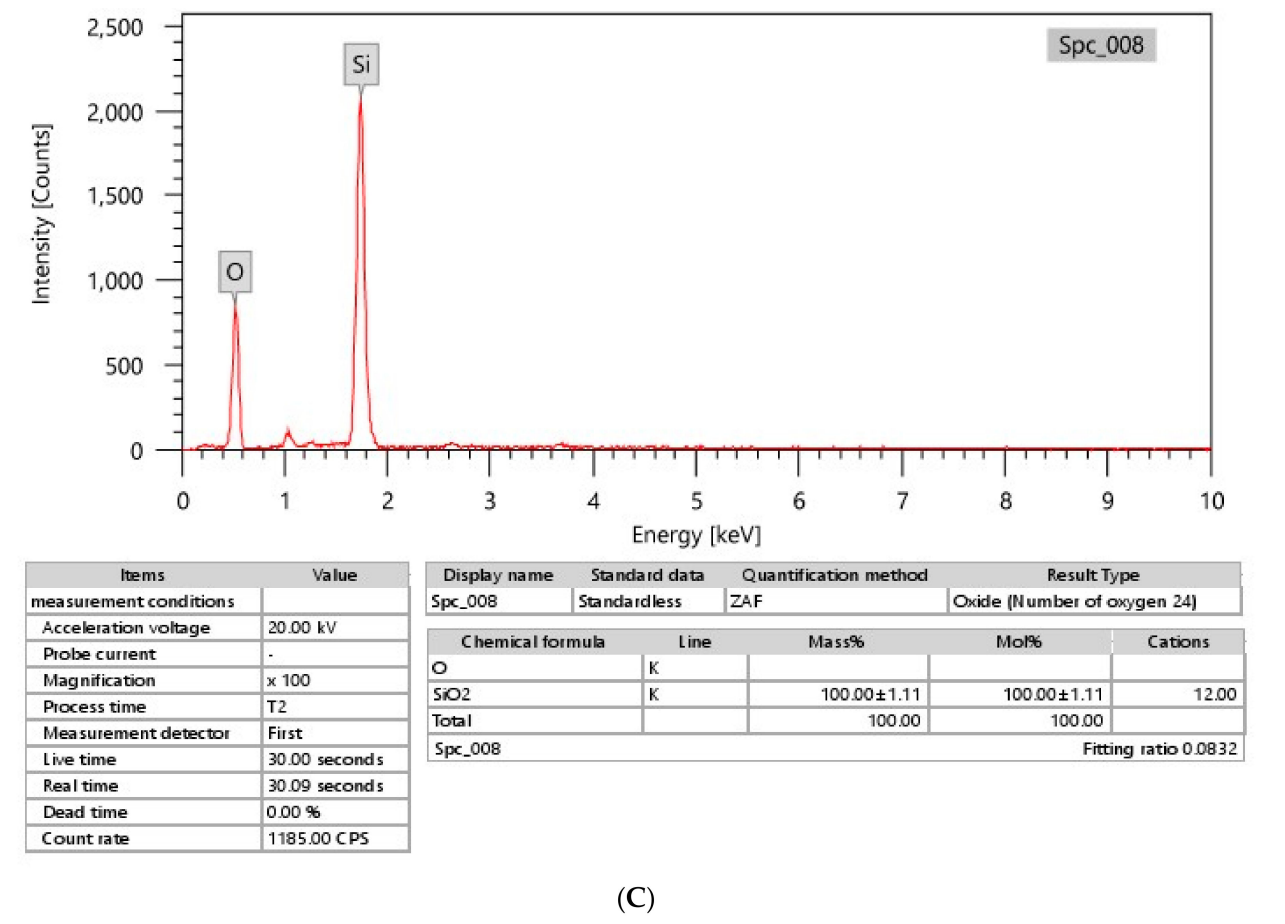
Characterization of the silicon dioxide nanoparticles (SiO_2_NPs) used in the current paper. (**A**) TEM image with an average size of 110.20 ± 3.28 nm. (**B**) The zeta potential and (**C**) the composition analysis (EDX) of the constituents, showing the presence of silicon and oxygen elements.

TEM images show the morphological information of the SiO_2_NPs used in the present study; an irregular spherical shape was found, with a relatively uniform size distribution (Figure 1A). The average size distribution of SiO_2_NPs was 110.20 ± 3.28 nm. The surface charge of SiO_2_NPs in water was measured as a zeta potential of −40.00 mV (Figure 1B). The spectroscopic composition analysis by EDX demonstrated silicon and oxygen elements in the constituents of the SiO_2_NP sample used in the present study (Figure 1C).

### 3.2. Serum Hepato-Renal Functions

No mortality was observed during the experimental period. Table 2 describes the alterations in serum biochemical parameters in the control and SiO_2_NP-exposed fish groups over 3 weeks. Pairwise comparisons with the control group showed a significant dose-dependent elevation in serum ALP, ALT, and AST enzyme activities, and higher blood urea and creatinine levels were evident in the SiO_2_NP-intoxicated fish groups (*p* < 0.05). The highest serum ALT, AST, and ALP activity and blood urea and creatinine levels were recorded in the fish group exposed to 100 mg SiO_2_NPs/L.

### 3.3. Gene Transcription Profile

#### 3.3.1. Gill Tissues

**Figure 2:**
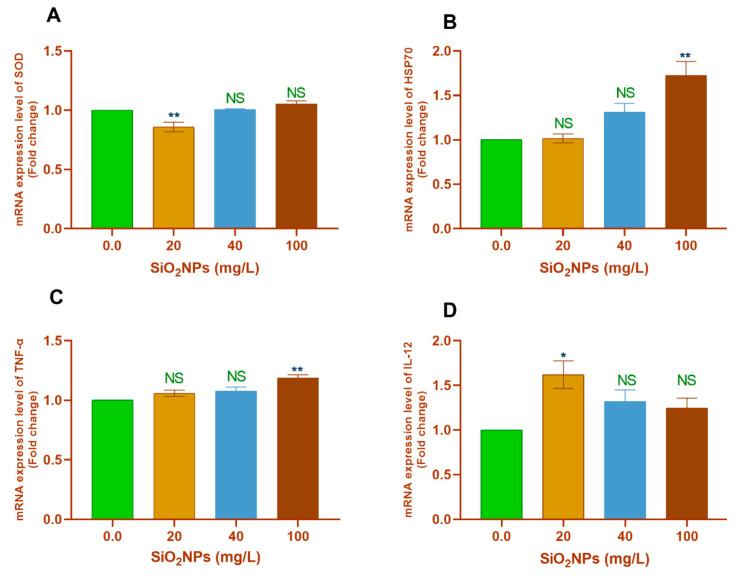
Transcription profile of (**A**) *SOD*, (**B**) *HSP70*, (**C**) *TNF-α*, and (**D**) *IL-12* genes in the gill tissues of Nile tilapia juveniles after exposure to different concentrations of SiO_2_NPs (0.0, 20, 40, and 100 mg/L) for 3 weeks. The values are expressed as mean ± SEM (*n* = 9). Asterisks (**) (*p* < 0.01) and (*) (*p* < 0.05) indicate significant differences between the exposure groups as compared with the control group. NS indicates non-significant differences.

With regard to the pairwise comparisons with the control group (0.0 mg SiO_2_NPs/L), significant downregulation of *SOD* gene transcription was observed in the 20 mg SiO_2_NPs/L group (*p* < 0.01; Figure 2A). Moreover, exposure to 20 mg/L SiO_2_NPs significantly upregulated the transcription of the *IL-12* gene (*p* < 0.05; Figure 2D).

**Figure 3:**
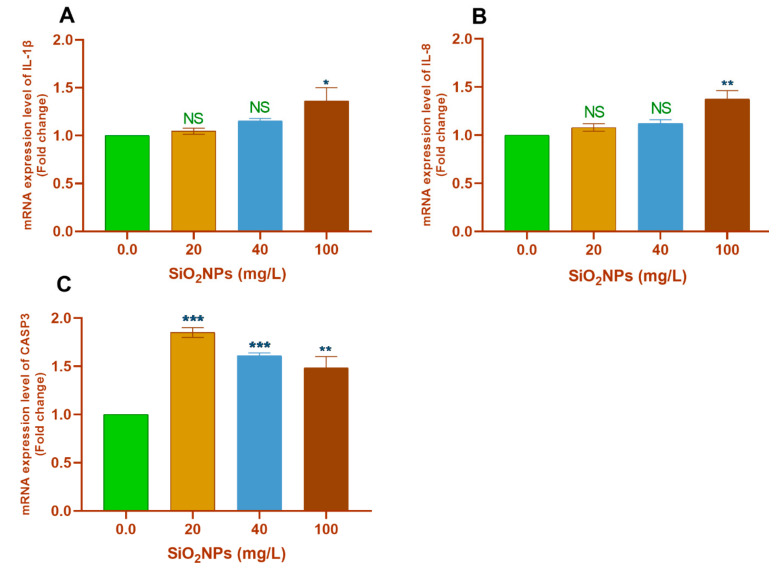
Transcription profile of the (**A**) *IL-1β*, (**B**) *IL-8*, (**C**) *CASP3* genes in gills of Nile tilapia juveniles after exposure to different concentrations of SiO_2_NPs (0.0, 20, 40, and 100 mg/L) for 21 days. The values are expressed as mean ± SEM (*n* = 9). Asterisks (*) (*p* < 0.05), (**) (*p* < 0.01), and (***) (*p* < 0.001) indicate significant differences between the exposure groups compared with the control group. NS indicates non-significant differences.

However, exposure to 100 mg/L SiO_2_NPs significantly upregulated the transcription of the *HSP70* (*p* < 0.01; Figure 2B), *TNF-α* (*p* < 0.01; Figure 2C), *IL-1β* (*p* < 0.05; Figure 3A), and *IL-8* (*p* < 0.01; Figure 3B) genes. Furthermore, all tested concentrations of SiO_2_NPs significantly upregulated the transcription of the *CASP3* gene (*p* < 0.05; Figure 3C).

On the other hand, exposure of fish to SiO_2_NPs did not cause any significant differences (*p* < 0.05) in the expression of *GPX*, *CAT*, and *IL-10* genes in the gill tissues (Supplementary Figure S1).

#### 3.3.2. Hepatic Tissues

**Figure 4:**
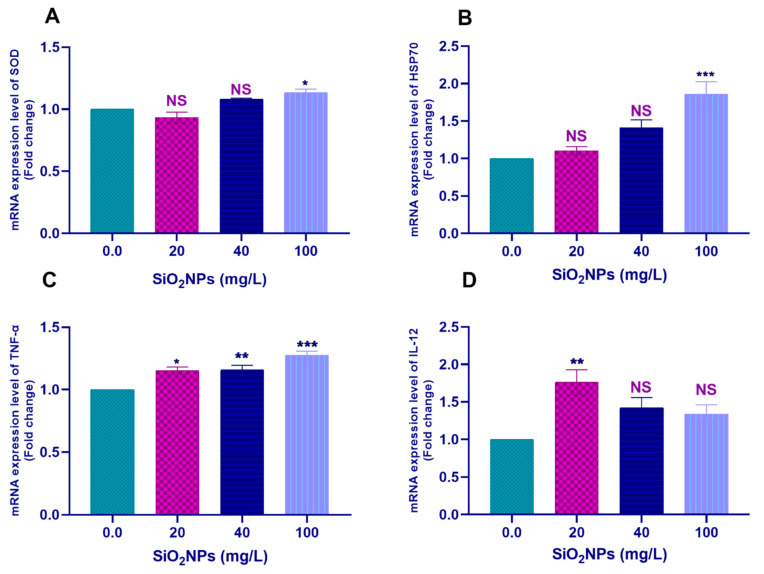
Transcription profile of the (**A**) *SOD*, (**B**) *HSP70*, (**C**) *TNF-α*, and (**D**) *IL-12* genes in the hepatic tissues of Nile tilapia juveniles after exposure to different concentrations of SiO_2_NPs (0.0, 20, 40, and 100 mg/L) for 21 days. The values are expressed as mean ± SEM (*n* = 9). Asterisks (*) (*p* < 0.05), (**) (*p* < 0.01), and (***) (*p* < 0.001) indicate significant differences between the exposure groups compared with the control group. NS indicates non-significant differences.

**Figure 5:**
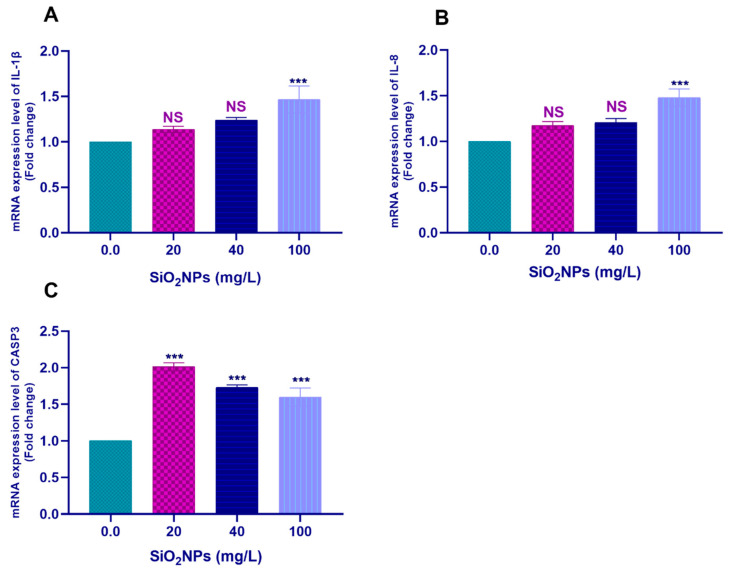
Transcription profile of the (**A**) *IL-1β*, (**B**) *IL-8*, (**C**) *CASP3* genes in the hepatic tissues of Nile tilapia juveniles after exposure to SiO_2_NPs at different concentrations (0.0, 20, 40, and 100 mg/L) for 21 days. The values are expressed as mean ± SEM (*n* = 9). Asterisks (***) (*p* < 0.001) indicate significant differences between the exposure groups compared with the control group. NS indicates non-significant differences.

Pairwise comparisons with the control group (0.0 SiO_2_NPs mg/L) showed that the exposure to 100 mg/L SiO_2_NPs significantly upregulated the transcription of *SOD* (*p* < 0.05; Figure 4A), *HSP70* (*p* < 0.001; Figure 4B), *IL-1β* (*p* < 0.01; Figure 5A), and *IL-8* (*p* < 0.001; Figure 5B) genes. Moreover, exposure to 20 mg/L SiO_2_NPs significantly upregulated the transcription of *IL-12* (*p* < 0.01; Figure 4D).

Remarkably, all tested concentrations of SiO_2_NPs significantly upregulated the transcription of the *TNF-α* (Figure 4C) and *CASP3* (Figure 5C) genes (*p* < 0.05).

On the other hand, exposure to SiO_2_NPs did not induce any significant differences (*p* < 0.05) in the mRNA expression values of *GPX*, *CAT*, and *IL-10* genes in the hepatic tissues of the exposed Nile tilapia (Supplementary Figure S2).

### 3.4. Histopathological Findings

#### 3.4.1. Hepatopancreas

**Figure 6:**
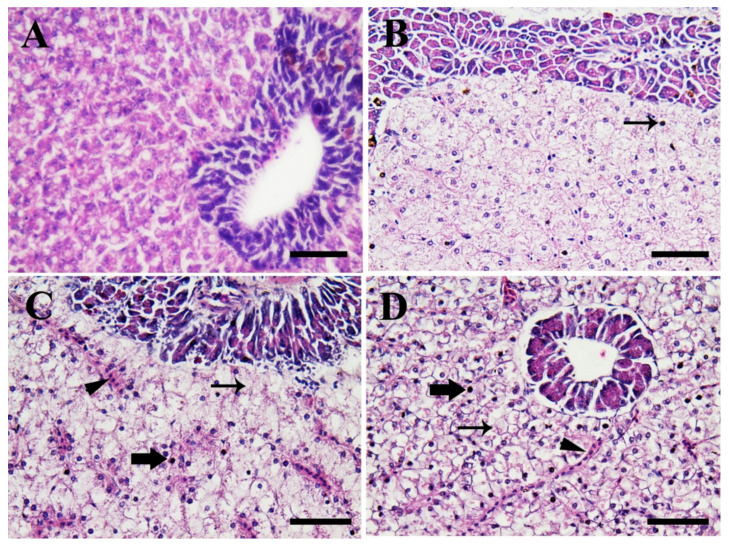
Illustrative photomicrographs of the hepatopancreatic tissues of Nile tilapia (hematoxylin and eosin (H & E) stain, scale bar = 50 μm) from the control group (**A**), and the fish groups exposed to SiO_2_NPs at 20 mg/L (**B**), 40 mg/L (**C**) and 100 mg/L (**D**), respectively, for 3 weeks. (**A**) Normal hepatopancreatic architecture, hepatic cord, and acini of the exocrine pancreas. (**B**) Slight fatty vacuolized and necrotic hepatocytes (arrow). (**C**) Moderate congestion (arrowhead), diffuse fatty vacuolized hepatocytes (thin arrow), moderate necrosis (thick arrow), and mononuclear inflammatory cell infiltrations in the pancreatic acinar cells. (**D**) Severe necrosis (thick arrow), fatty degeneration of hepatocytes (thin arrow), and severe congestion of the blood sinusoids (arrowhead).

Figure 6 shows the photomicrographs in the hepatic tissues of Nile tilapia in the control group compared to fish groups exposed to SiO_2_NPs for 3 weeks. Fish in the control group (0.0 SiO_2_NPs mg/L) (Figure 6A) showed normal hepatopancreatic architecture, hepatic cord, and acini of the exocrine pancreas. Meanwhile, fish exposed to 20 mg/L SiO_2_NPs (Figure 6B) showed slightly fatty vacuolized and necrotic hepatocytes. Moreover, fish exposed to 40 mg/L SiO_2_NPs (Figure 6C) showed moderate congestion, diffuse fatty vacuolized hepatocytes, and moderate necrosis. The hepatocytes contained an eccentrically located necrotic nucleus and mononuclear inflammatory cell infiltrations, predominantly in the pancreatic acinar cells. Furthermore, fish exposed to 100 mg/L SiO_2_NPs (Figure 6D) showed severe necrosis, fatty degeneration of hepatocytes, and severe blood sinusoid congestion.

#### 3.4.2. Gills

**Figure 7:**
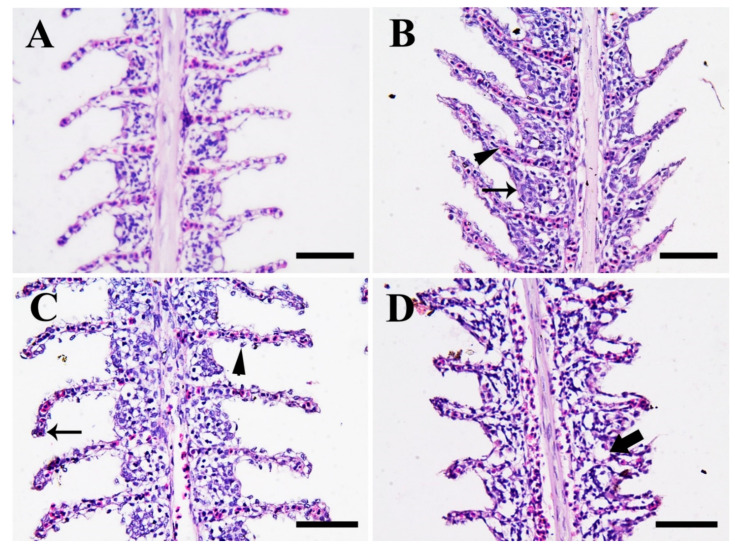
Illustrative photomicrographs of the gills of Nile tilapia (H & E stain, scale bar = 50 μm) from the control group (**A**), and fish groups exposed to SiO_2_NPs at 20 mg/L (**B**), 40 mg/L, (**C**) and 100 mg/L (**D**), respectively, for 3 weeks. (**A**) Normal gill architecture with normal primary and secondary lamellae. (**B**) Slight congestion of primary and secondary lamellae (arrowhead) and slight epithelial hyperplasia (arrow). (**C**) Moderate epithelial necrosis, desquamation, epithelial layer rupture (arrowhead), and telangiectasis of the secondary lamellae (arrow). (**D**) Severe fusion of secondary lamellae, necrosis, and rupture of the epithelial layer (arrow).

Figure 7 shows photomicrographs in the gill tissues of Nile tilapia in the control group compared to fish groups exposed to SiO_2_NP for 3 weeks. Fish in the control group (0.0 SiO_2_NPs mg/L) (Figure 7A) showed normal gill architecture with normal primary and secondary lamellae. Meanwhile, fish exposed to 20 mg/L SiO_2_NPs (Figure 7B) showed slight congestion of primary and secondary lamellae and slight epithelial hyperplasia. Moreover, fish exposed to 40 mg/L SiO_2_NPs (Figure 7C) showed moderate epithelial necrosis, desquamation, epithelial layer rupture, and telangiectasis of the secondary lamellae. Furthermore, fish exposed to 100 mg/L SiO_2_NPs (Figure 7D) displayed a severe fusion of secondary lamellae, necrosis, and rupture of the epithelial layer.

#### 3.4.3. Posterior Kidney

**Figure 8:**
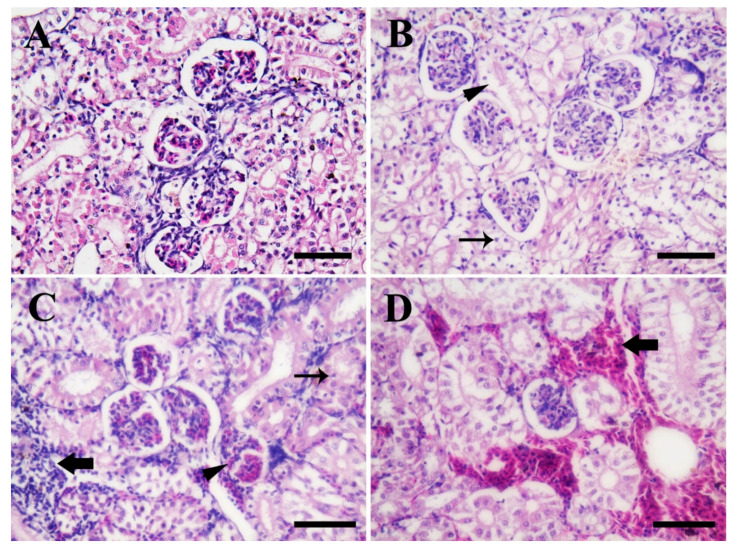
Illustrative photomicrographs of the posterior kidneys of Nile tilapia (H & E stain, scale bar = 50 μm) from the control group (**A**), and fish groups exposed to SiO_2_NPs at 20 mg/L (**B**), 40 mg/L (**C**), and 100 mg/L (**D**), respectively, for 3 weeks. (**A**) Normal renal architecture. (**B**) Slight necrosis of several renal tubules and edema (arrowhead), as well as pyknotic nuclei (arrow). (**C**) Moderate necrosis of some renal tubules and edema (thin arrow), congested glomeruli (arrowhead), and extensive filtration of inflammatory cells (thick arrow). (**D**) Extensive inter-tubular hemorrhages (arrow).

Figure 8 shows the photomicrographs in the posterior kidney tissues of Nile tilapia in the control group compared to fish groups exposed to SiO_2_NPs for 3 weeks. Fish in the control group (0.0 SiO_2_NPs mg/L) (Figure 8A) showed normal renal architecture (renal tubules, lining epithelium, glomerulus, and Bowman’s spaces). Meanwhile, fish exposed to 20 mg/L SiO_2_NPs (Figure 8B) showed slight necrosis, edema, and evident pyknotic nuclei of several renal tubules. Moreover, fish exposed to 40 mg/L SiO_2_NPs (Figure 8C) showed moderate necrosis of some renal tubules, edema, congested glomeruli, and evident and extensive filtration of inflammatory cells. Furthermore, fish exposed to 100 mg/L SiO_2_NPs (Figure 8D) showed extensive inter-tubular hemorrhages.

## 4. Discussion

### 4.1. Hepato-Renal Functions

ALT and AST are liver enzymes that are considered as significant biomarkers for liver functions. Their release into the bloodstream and elevation of their serum levels are indicators for the occurrence of liver problems such as disease or damage to fish hepatic tissues following exposure to aquatic toxicants [36]. ALP plays a significant role in the metabolism within the liver, and its elevated concentrations in the bloodstream can be used as a biomarker of hepatitis and hepatic damage [37]. Moreover, the elevation of the kidney function indices such as urea and creatinine might be linked to the impairment of tubular functions and glomerular insufficiency [38].

In the present investigation, significant dose-dependent elevations in serum ALP, ALT, and AST enzyme activities as well as higher blood urea and creatinine levels in SiO_2_NP-intoxicated fish groups were found after long-term exposure (3 weeks). Therefore, these outcomes suggested that exposure of fish to sub-lethal concentrations of SiO_2_NPs induced hepato-renal failure and damage to the hepatic and renal tissues. Parallel to our findings, it was found that the exposure of Mozambique tilapia to sub-lethal levels of SiO_2_NPs (60, 100, and 140 mg/L) for 96 h induced considerable elevation of hepatic AST and ALT enzyme activities [16] and a dose-dependent increase in urea and creatinine levels as compared to fish in the control group [17]. Similarly, elevated ALT and AST activities were found in *Pangasius hypophthalmus* exposed to selenium NPs [39]. Moreover, elevated ALT, AST, and ALP enzyme activities and urea and creatine values were noted in Nile tilapia exposed to zinc oxide NPs (ZnONPs) [5] or green synthesized silver NPs [40].

### 4.2. Gene Transcription Profile

Herein, Nile tilapia exposed to 100 mg/L SiO_2_NPs showed significantly upregulated transcription of the *SOD*, *HSP70*, *TNF-α*, *IL-1β*, and *IL-8* genes. Moreover, exposure to 20 mg/L SiO_2_NPs significantly upregulated the transcription of *IL-12* as compared to controls. Further detailed studies should be demonstrated in order to identify why the significant upregulation if *IL-12* gene was noted at the lowest dose concentrations only. The expression of these genes in fish tissues defines the toxicological effects on the exposed fish. Expression of the *HSP70* gene indicates that fish were stressed after SiO_2_NP exposure. Notably, it has been reported that the expression of *HSP70* gene is closely related to fish exposure to stressors [41,42,43]. Expression of *TNF-α*, *IL-1β*, and *IL-8* genes in fish tissues is an indicator of inflammatory signs occurring in exposed tissues. In this regard, it was previously shown that tumor necrosis factors such as *TNF-α* are implicated in inflammation, apoptosis, and cell proliferation [44]. Moreover, pro-inflammatory cytokines such as *IL-1β*, *IL-8*, *TNF-α,* and *IL-12* are defined as important molecules involved in hematopoiesis as well as inflammatory and adaptive immune responses in fish [45,46,47,48].

The expression of *SOD* gene in liver and gills of Nile tilapia SiO_2_NPs exposure indicates that fish were exposed to oxidative stress. Oxidative stress is a state of inequality between the endogenous antioxidant mechanisms of the host body and the overproduction of ROS such as hydrogen peroxides (H_2_O_2_) and superoxide (O_2_^−^) following exposure to xenobiotics [49]. The enzymatic endogenous antioxidative mechanisms that help in ROS attenuation include CAT, SOD, and GPX enzymes [50]. SOD enzyme catalyzes the dismutation of O_2_^−^ radicals into the ordinary oxygen (O_2_) and H_2_O_2_. Meanwhile, the CAT enzyme catalyzes the conversion of H_2_O_2_ to H_2_O [51]. Furthermore, selenium-dependent GPX enzymes act by catalyzing the conversion of H_2_O_2_ and fatty acid hydro-peroxides into water and fatty acid alcohol through reduced glutathione (GSH), and thus help in the protection of the cell membranes against the oxidative damage [52].

Zhu et al. [23] reported substantial upregulation of the mRNA expression of the *SOD1* gene in zebrafish larvae exposed to 100 mg/L SiO_2_NPs for 5 days. Similarly, increased transcription of the *SOD1* gene was found in zebrafish embryos exposed to ZnONPs [53]. Fırat and Bozat [54] reported a significant increase in CAT, GPX, glutathione reductase, and glutathione-S-transferase enzyme activities in the gill tissues of Nile tilapia exposed to titanium dioxide NPs (TiO_2_NPs) for 14 days.

Consistent with our results, significant upregulation of *TNF-α*, *IL-1β*, and *IL-6* genes has been reported in the spleen of zebrafish exposed to 50 mg/L graphene oxide [55]. Likewise, increased transcription of *TNF-α*, *IL-1β*, *HSP70*, and *IL-6* genes was observed in the gills of *Sparus aurata* exposed to gold nanoparticles [56]. Furthermore, dose-dependent increases in the mRNA expression values of *TNF-α* and *IL-1β* genes in the intestines of *Epinephelus coioides* juveniles exposed to copper oxide NPs (CuONPs) have been reported [57].

In the present study, all tested concentrations of SiO_2_NPs significantly upregulated the transcription of the *CASP3* gene in liver and gills of the exposed Nile tilapia. Caspases are important mediators of apoptosis (programmed cell death) [58]. Moreover, Jänicke et al. [59] illustrated that *CASP3* is required for DNA damage and several morphological alterations associated with apoptosis. Thus, these findings suggest that exposure of fish to SiO_2_NPs induced apoptotic changes in the affected hepatocytes and gill epithelial cells. Consistent with our results, increased transcription of *CASP3* and *CASP9* was reported in in *Takifugu fasciatus* juveniles exposed to copper oxide NPs [60]. Likewise, significant upregulation of *CASP3*, *CASP8*, and *CASP9* was also noted in the gill tissues of *Oryzias latipes* exposed to multiwall carbon nanotubes (MWCNTs) [61].

### 4.3. Histopathological Alterations

Histopathological alterations in fish tissues are an indicator of exposure to aquatic pollutants [35,62,63,64]. Herein, the exposure of Nile tilapia to several sub-lethal concentrations of SiO_2_NPs for 3 weeks resulted in varying degrees of fatty vacuolation, necrosis, congestion, and fatty degeneration in hepatocytes, as well as the congestion of hepatic sinusoids in relation to exposure dosage. Degenerative changes were also recorded in the hepatocytes of Mozambique tilapia exposed to SiO_2_NPs [13,16,20]. Similarly, Kaya et al. [65] described how sub-chronic exposure of Nile tilapia to ZnONPs resulted in vacuolation and blood sinusoid congestion. Furthermore, Murali et al. [66] demonstrated hepatic damage in Mozambique tilapia exposed to sub-lethal concentrations of aluminum oxide NPs. Besides, Kumar et al. [39] exemplified how acute exposure of *P. hypophthalmus* to selenium NPs induced vacuolation, necrosis, pyknotic nuclei, and interstitial edema of the hepatopancreatic cells. Of note, it has been found that exposure of fish to toxicants and aquatic pollutants may cause disorganization of the hepatocytes, changes in the size and shape of nuclei, and focal necrosis [63,67].

The exposure of Nile tilapia to different sub-lethal SiO_2_NPs concentrations for 3 weeks resulted in varying degrees of congestion, epithelial hyperplasia, necrosis, desquamation, rupture, and telangiectasis in the primary and secondary gill lamellae depending on the exposure doses. Vidya and Chitra [13] showed that exposure of Mozambique tilapia to sub-lethal dose SiO_2_NPs induced uplifting of gill epithelium, damage and vacuolization in the gill arches, aneurysm, and curling of secondary gill lamellae. In a similar sense, epithelial hyperplasia, aneurism, and epithelial lifting were recorded in the secondary gill lamellae of Nile tilapia exposed to MWCNTs [68] or ZnONPs [69]. Moreover, thickening of gill lamellae and edematous changes were observed in common carp (*Cyprinus carpio*) juveniles exposed to a sub-lethal dose of TiO_2_NPs [70].

The exposure of Nile tilapia to different sub-lethal concentrations of SiO_2_NPs for 3 weeks resulted in varying degrees of necrosis, edema, hemorrhage, pyknotic nuclei, edema, and congestion in the renal glomeruli and renal tubules depending on the exposure doses. Consistent with our results, Athif et al. [17] demonstrated that exposure of Mozambique tilapia to sub-lethal doses of SiO_2_NPs for 96 h induced vacuolation of the proximal and distal tubules, hemorrhage, and degeneration of the renal tubules. Likewise, degenerative changes in the renal tubules, pyknotic nuclei, and structural disarray in the kidney tissues were reported in common carp exposed to CuONPs [71]. Congestion, degeneration in the renal tubules, and pigment accumulation in the intratubular spaces were noted in common carp exposed to chronic dietary ZnONP toxicity [72]. Moreover, necrosis, cytoplasmic vacuolations, and deformations of the renal tubules were recorded in Nile tilapia exposed to ZnONPs [69].

## 5. Conclusions

Our findings illustrated that Nile tilapia exposure to sub-lethal concentrations of SiO_2_NPs (20, 40, and 100 mg/L) induced serum biochemical changes, histopathological alterations, and modulation of the gene transcription profile during long-term exposure. Taken together, the increased liver and kidney dysfunction indicator values, irreversible degenerative alterations in hepatopancreatic, renal, and gill tissues, and upregulation of transcription of *HSP70*, *CASP3*, pro-inflammatory cytokines, and oxidative stress-related genes provide strong evidence of the inflammatory responses, stress effects, and toxic effects of SiO_2_NPs. The current investigation also presents the first comprehensive overviews of the toxic effects of SiO_2_NPs at the molecular level, which may contribute positively to knowledge on SiO_2_NP toxicity in exposed aquatic organisms. Besides, these findings provide new and useful and reference insights for future research. Finally, taking into account the continuous and increasing demand for SiO_2_NP-based industrial products, their release into the aquatic ecosystems and the toxic impacts to exposed fish should be carefully considered.

## Figures and Tables

**Table 1:** Primer sequences, accession numbers, and primer efficiencies of the target mRNA used for the RT-PCR study.

Target mRNA	Primer Sequences (F: Forward, R: Reverse)	NCBI GenBank Accession No.	Slope	Efficiency %
***SOD***	F: 5′-CCCTACGTCAGTGCAGAGAT-3′	JF801727.1	−3.51	92.708%
	R: 5′-GTCACGTCTCCCTTTGCAAG-3′			
***GPX***	F: 5′-CGCCGAAGGTCTCGTTATTT-3′	NM_001279711.1	−3.55	91.289%
	R: 5′-TCCCTGGACGGACATACTT-3′			
***CAT***	F: 5′-CCCAGCTCTTCATCCAGAAAC-3′	JF801726.1	−3.41	96.451%
	R: 5′-GCCTCCGCATTGTACTTCTT-3′			
***HSP70***	F: 5′-CATCGCCTACGGTCTGGACAA-3′	FJ207463.1	−3.60	89.574%
	R: 5′-TGCCGTCTTCAATGGTCAGGAT-3′			
***TNF-α***	F: 5′-GGAAGCAGCTCCACTCTGATGA-3′	JF957373.1	−3.33	99.664%
	R: 5′-CACAGCGTGTCTCCTTCGTTCA-3′			
***IL-1β***	F: 5′-CAAGGATGACGACAAGCCAACC-3′	XM_003460625.2	−3.65	87.92%
	R: 5′-AGCGGACAGACATGAGAGTGC-3′			
***IL-8***	F: 5′-TCATTGTCAGCTCCATCGTG-3′	NM_001279704.1	−3.62	88.905%
	R: 5′-CCTGTCCTTTTCAGTGTGGC-3′			
***CASP3***	F: 5′-GGCTCTTCGTCTGCTTCTGT-3′	GQ421464.1	−3.36	98.435%
	R: 5′-GGGAAATCGAGGCGGTATCT-3′			
***IL-10***	F: 5′-CTGCTAGATCAGTCCGTCGAA-3′	XM_003441366.2	−3.44	95.298%
	R: 5′-GCAGAACCGTGTCCAGGTAA-3′			
***IL-12***	F: 5′-GGGTGCGAGTCAGCTATGAG-3′	XM_003437924.4	−3.59	89.912%
	R: 5′-GGTTGTGGATTGGTTGCGTC-3′			
***β-actin***	F: 5′-CCACACAGTGCCCATCTACGA-3′	EU887951.1	−3.522	92.278%
	R: 5′-CCACGCTCTGTCAGGATCTTCA-3′			

*SOD*: superoxide dismutase, *HSP70*: heat shock protein 70, *GPX*: glutathione peroxidase, *CAT*: catalase, *TNF-α*: tumor necrosis factor alpha, *CASP3*: caspase 3, *IL-1β*: interleukin 1 beta, *IL-8*: interleukin 8, *IL-10*: interleukin 10, *IL-12*: interleukin 12, *β-actin*: beta actin.

**Table 2:** Liver and kidney functions indices in the serum of Nile tilapia juveniles after exposure to different sub-lethal concentrations of SiO_2_NPs.

Parameters	SiO_2_NP Concentrations			
	Control (0.0 mg/L)	20 mg/L	40 mg/L	100 mg/L
**Liver function enzymes**				
**AST (U/L)**	44.91 ± 0.88 d	51.84 ± 2.00 c	61.23 ± 2.30 b	84.27 ± 1.50 a
**ALT (U/L)**	13.28 ± 0.61 c	14.28 ± 0.99 b	16.95 ± 1.35 b	22.61 ± 1.51 a
**ALP (U/L)**	9.62 ± 0.78 b	11.17 ± 1.20 ab	11.20 ± 0.58 ab	15.34 ± 1.56 a
**Kidney function indices**				
**Urea (mg/dL)**	6.67 ± 0.55 c	9.33 ± 0.61 b	11.42 ± 1.04 b	16.11 ± 0.86 a
**Creatinine (mg%)**	0.66 ± 0.06 c	1.06 ± 0.13 b	1.12 ± 0.07 ab	1.65 ± 0.36 a

Data represent means ± SE. Means with different letters in the same row are significantly different at *p* < 0.05. AST: aspartate transaminase, ALT: alanine transaminase, ALP: alkaline phosphatase.

## Data Availability

Accessible from the corresponding author on fair call.

## Supplementary Materials

The following are available online at https://www.mdpi.com/2079-7737/10/3/183/s1, Figure S1: Transcription profile of (A) GPX, (B) IL-10, and (C) CAT genes in the gill tissues of Nile tilapia juveniles after exposure to different concentrations of SiO2NPs (0.0, 20, 40, and 100 mg/L) for 3 weeks. Values are expressed as mean ± SEM (n = 9). NS indicates non-significant differences. Figure S2: Transcription profile of (A) GPX, (B) IL-10, and (C) CAT genes in liver of Nile tilapia juveniles after exposure to different concentrations of SiO2NPs (0.0, 20, 40, and 100 mg/L) for 3 weeks. Values are expressed as mean ± SEM (n = 9). NS indicates non-significant differences.

## Animal Welfare Statement

This experiment was conducted under the guidelines of the Faculty of Veterinary Medicine, Alexandria University, Alexandria, Egypt.

## Appendix A

We have followed the following quality control measures in RT-PCR during the present study:

The quality of the extracted RNA was confirmed with 2 % agarose electrophoresis.

The quality and quantity of the extracted RNA was determined by Nanodrop (Quawell, USA) samples of 1.8 or more A260/A280 RNA were used.

Ensure no contamination: use distinct areas for sample preparation, PCR setup, and post-PCR analysis.

To avoid contamination from old amplicons, we set up the stations on separate benchtops, one for pre-PCR (for PCR reaction setup only) and the other for post-PCR (purifying PCR-amplified DNA, measuring DNA concentration, running agarose gels, and analyzing PCR products).

Keep the PCR machine and electrophoresis apparatus in the post-PCR area.

Prepare and store reagents for PCR separately and use them solely for their designated purpose.

Aliquot reagents in small portions and store them in either location based on their use in pre-PCR or post-PCR applications. Store the aliquots separately from other DNA samples.

Use separate sets of pipettes and pipette tips, lab coats, glove boxes, and waste baskets for the pre-PCR and post-PCR areas. If you are doing NGS library prep, use a surface decontaminant for nucleic acids to wipe down benchtops and pipettes before you begin.

Use pipettes and pipette tips with aerosol filters dedicated for DNA sample and reaction mixture preparation.

Follow the golden rule of PCR: DO NOT bring any reagents, equipment, or pipettes used in a post-PCR area back to the pre-PCR area.

Keep the number of PCR cycles to a minimum, as highly sensitive assays are more prone to the effects of contamination.

To avoid primer dimers: we optimize the reaction.
