# Supplementary material for: Hazardous Effects of SiO2 Nanoparticles on Liver and Kidney Functions, Histopathology Characteristics, and Transcriptomic Responses in Nile Tilapia (Oreochromis niloticus) Juveniles
Source: Biology (Basel). 2021 Mar 2;10(3):183. doi: 10.3390/biology10030183 (PMC8000872; doi:10.3390/biology10030183)
Supplement: Supplementary file 1 [file biology-10-00183-s001.pdf]

biology

MDPI

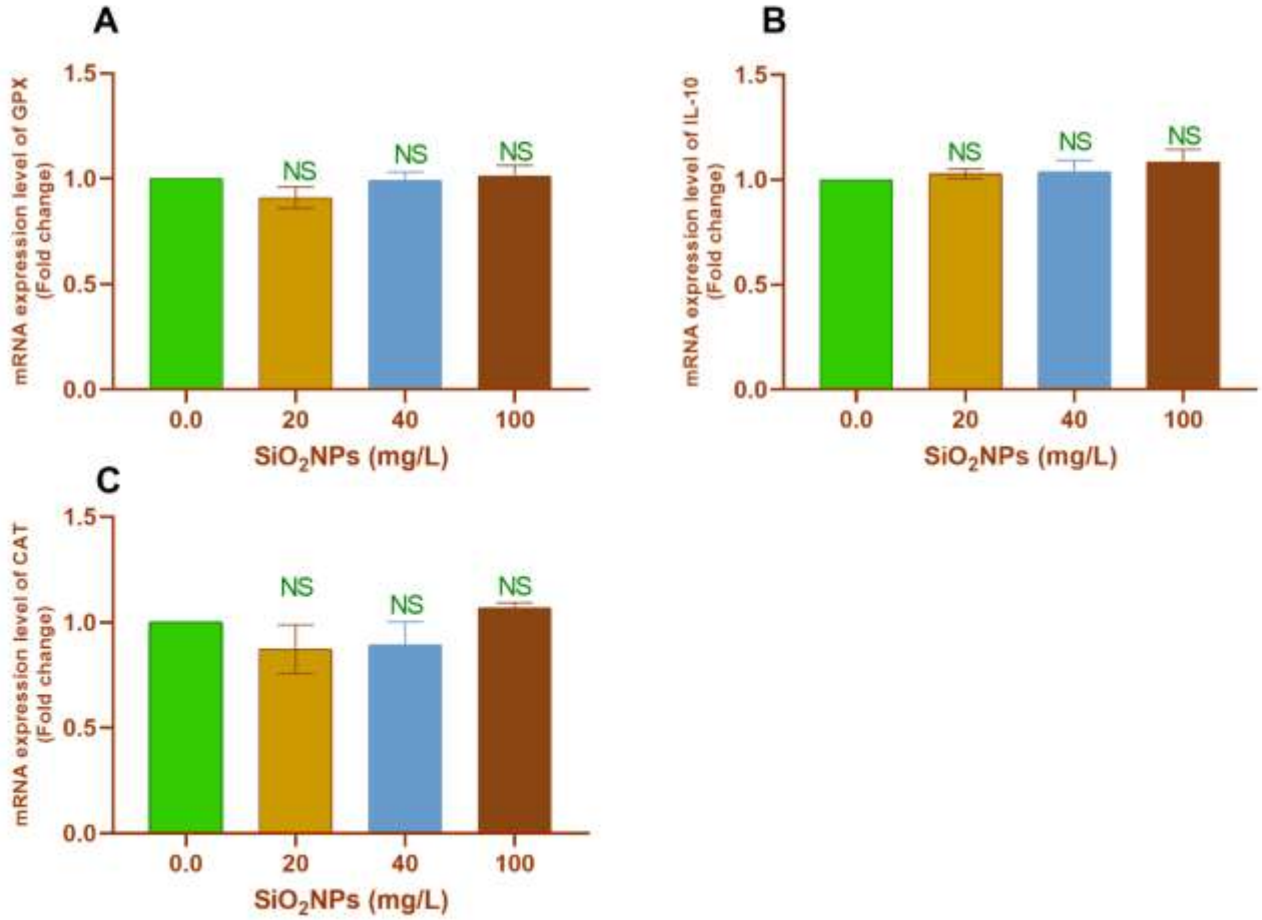

Figure S1. Transcription profile of (A) *GPX*, (B) *IL-10*, and (C) *CAT* genes in the gill tissues of Nile tilapia juveniles after exposure to different concentrations of SiO_2NPs (0.0, 20, 40, and 100 mg/L) for 3 weeks. Values are expressed as mean ± SEM (n = 9). NS indicates non-significant differences.

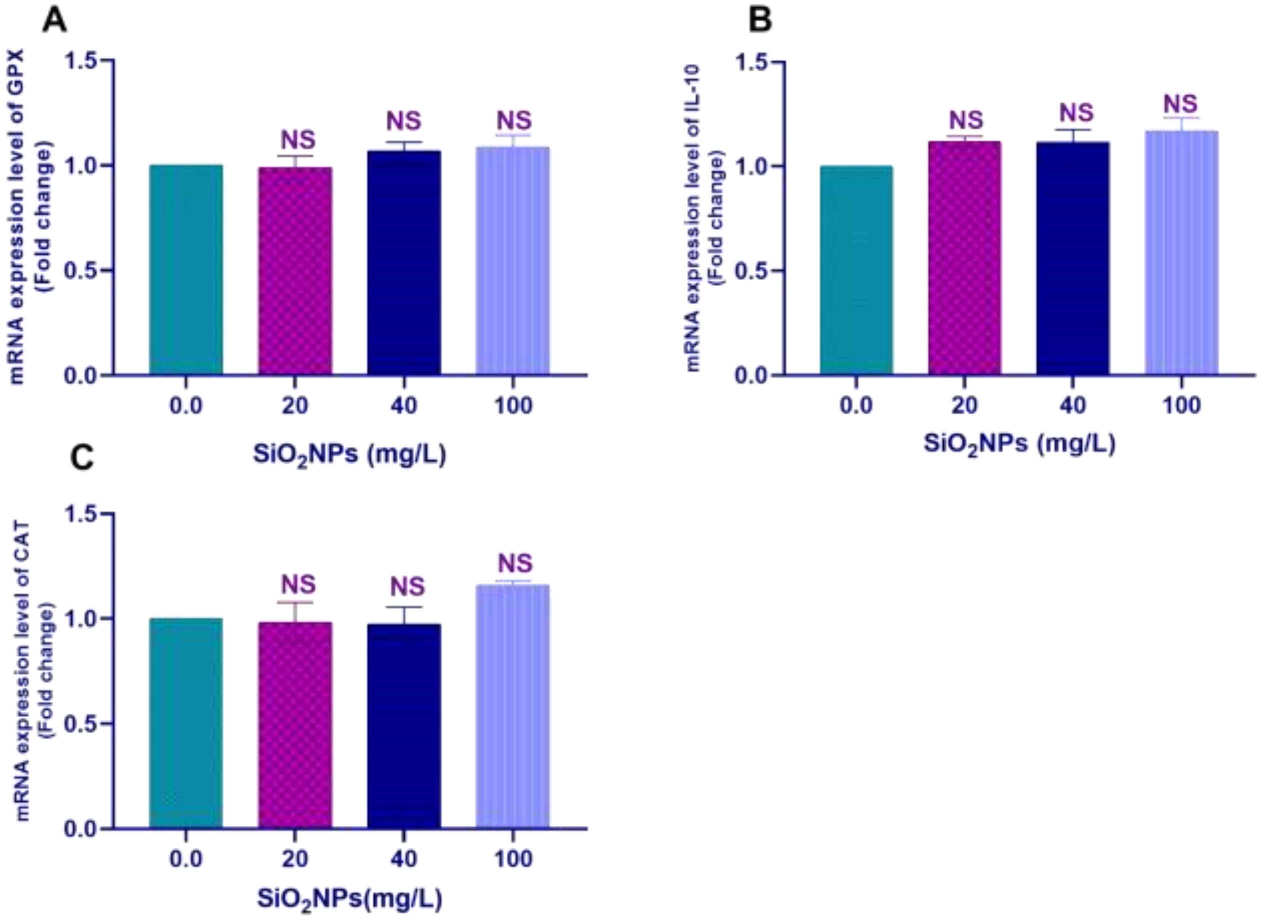

Figure S2 Transcription profile of (A) *GPX*, (B) *IL-10*, and (C) *CAT* genes in liver of Nile tilapia juveniles after exposure to different concentrations of SiO_2NPs (0.0, 20, 40, and 100 mg/L) for 3 weeks. Values are expressed as mean ± SEM (n = 9). NS indicates non-significant differences.
